# EEG Biomarkers Differentiating Alzheimer's Disease and Amyloid‐Negative Controls

**DOI:** 10.1002/alz70856_105594

**Published:** 2026-01-08

**Authors:** Nayoung Ryoo, Young Ho Park, SangYun Kim

**Affiliations:** ^1^ Eun‐pyeong St. Mary's Hospital of the Catholic University of Korea, Seoul, Seoul, Korea, Republic of (South); ^2^ Seoul National University Bundang Hospital, Seongnam, Korea, Republic of (South); ^3^ Seoul National University Bundang Hospital, Seoul National University College of Medicine, Seongnam, Gyeonggi‐do, Korea, Republic of (South)

## Abstract

**Background:**

Electroencephalography (EEG) is a cost‐effective and non‐invasive tool for evaluating functional brain changes in Alzheimer's disease (AD). While traditional diagnostic methods such as amyloid PET and MRI are valuable, they are often expensive and impractical for routine clinical use. This study aimed to identify specific EEG biomarkers that differentiate individuals with Alzheimer's dementia (AD) from cognitively normal (CN) controls and assess their diagnostic utility using machine learning techniques.

**Method:**

A total of 58 CN controls with amyloid PET negativity and 36 individuals with AD with amyloid PET positivity were recruited. Age and gender adjustments were applied to select 33 participants from each group for analysis. Resting‐state EEG data were recorded under eyes‐closed conditions. Power spectral density and graph‐theory‐based network connectivity metrics were extracted for delta, theta, alpha, and beta frequency bands. Machine learning classification using Diagonal Linear Discriminant Analysis (DLDA) was performed to evaluate the diagnostic performance of EEG features, both individually and in combination.

**Result:**

The AD group exhibited significantly increased global theta power and higher relative power in both low and high beta bands compared to the CN group. Diagnostic accuracy using power spectral features alone reached 90.91%, with a sensitivity of 87.88% and a specificity of 93.94%. When network connectivity metrics alone were used, the accuracy was 84.85%, with a sensitivity of 75.76% and a specificity of 93.94%. Combining power spectral and network connectivity features improved the diagnostic accuracy to 93.94%, with a sensitivity of 90.91% and a specificity of 96.97%.

**Conclusion:**

This study demonstrates that EEG biomarkers, particularly global theta and beta power and network connectivity metrics, are effective in distinguishing Alzheimer's dementia from cognitively normal controls confirmed by amyloid PET status. Combining power and network connectivity features enhances diagnostic performance, underscoring the potential of EEG as a practical and non‐invasive biomarker for Alzheimer's disease.

